# Isometric exercise in cardiac magnetic resonance imaging: an initial experience using fast imaging

**DOI:** 10.1186/1532-429X-13-S1-P386

**Published:** 2011-02-02

**Authors:** Kristian H Mortensen, Jennifer A Steeden, Joseph Panzer, Andrew M Taylor, Vivek Muthurangu

**Affiliations:** 1 UCL Institute of Child Health, London, UK; 2 UCL Department of Medical Physics & Bioengineering, London, UK

## Introduction

Isometric exercise is a powerful stimulator of the cardiovascular system. Therefore, exercise can be used to unmask subtle cardiovascular changes that are not evident at rest. MRI is a proven method for comprehensive cardiovascular assessment in the resting state. Unfortunately, lack of fast imaging sequences and an appropriate exercise test has prohibited the use of isometric exercise in MRI.

## Purpose

We aimed to devise an isometric exercise test for use in MRI in order to comprehensively assess the hemodynamic response to exercise using fast-imaging MRI.

## Methods

A sustained isometric biceps exercise test was devised for the MRI scanner. Ten healthy male volunteers (24 to 44 years) underwent three minutes of sustained isometric exercise using a weight load adjusted to 35% of maximum biceps strength. The hemodynamic response was elucidated during rest, exercise [1, 2 and 3 minutes] and recovery [1, 3 and 5 minutes]. At each time point we obtained 1) aortic flow - spiral prospectively-gated breath-hold phase contrast CMR, 2) LV volumes - real-time radial k-t sensitivity encoding free-breathing CMR and 3) oscillometric upper arm blood pressure. Using this data vascular resistance and compliance was calculated.

## Results

**Figure 1:**
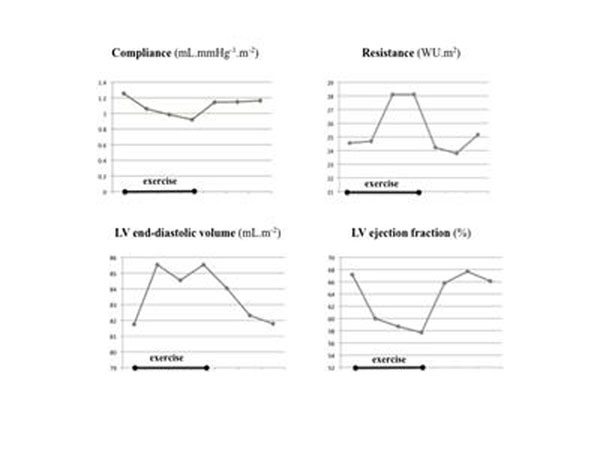
Mean cardiovascular measures during rest, isometric exercise and recovery in 10 healthy male volunteers.

All participants completed the isometric exercise test. All data sets were complete. Sustained isometric exercise in the MRI scanner produced a marked hemodynamic response (Table 1). Both vascular and left ventricular measures were changed by the exercise. Most indices were changed at one minute, while all were changed after two and three minutes (Figure 1). The return towards the resting state at recovery was fast (Table 1). There was no evidence of compensatory adaptive hemodynamic mechanisms during the recovery period in the cardiovascular measures.

**Table 1:** Mean (SD) cardiovascular measures during rest, isometric exercise and recovery

	***Rest***	***Isometric biceps exercise***			***Recovery***		
		*1 min*	*2 min*	*3 min*	*1 min*	*3 min*	*5 min*

***Systolic blood pressure** (mmHg)*	111 (10)	128 (16) **†**	140 (21) **†**	145 (17) **†**	116 (13) **†**	113 (12)	112 (11)
***Heart rate** (beats.min^-1^)*	64 (9)	80 (8)**†**	81 (9)**†**	82 (13)**†**	65 (8)	64 (9)	63 (9
***Stroke volume** (mL.m^-2^)*	54 (8)	51 (9)**†**	49 (9)**†**	49 (8)**†**	55 (8)	56 (9)	54 (8)
***Cardiac output** (L.min^-1^.m^-2^)*	3.4 (0.5)	4.1 (0.8)**†**	4.0 (0.8)**†**	4.0 (0.7)**†**	3.6 (0.4)	3.6 (0.5)	3.4 (0.4)
***Compliance** (mmHg.mL^-1^.m^-1^)*	1.3 (0.3)	1.1 (0.2)**†**	1.0 (0.2)**†**	0.9 (0.3)**†**	1.1 (0.2)	1.1 (0.3)	1.2 (0.2)
***Resistance** (WU.m^2^)*	24.6 (3.4)	24.7 (4.6)	28.1 (6.6)**†**	28.1 (4.9)**†**	24.2 (3.3)	23.8 (3.2)	25.1 (3.4)
***End-diastolic volume** (mL.m^-2^)*	82 (12)	86 (16)**†**	85 (15)**†**	86 (14)**†**	82 (13)	82 (13)	82 (14)
***Ejection fraction** (%)*	67 (9)	60 (9)**†**	58 (9)**†**	58 (6)**†**	66 (8)	68 (6)	66 (5)

## Conclusion

Isometric biceps exercise testing is highly feasible using a MRI safe methodology, which allows exercise in the supine position within the scanner. The test evokes a hemodynamic response that corresponds to isometric exercise conducted outside the MRI scanner. The hemodynamic response can be comprehensively and safely assessed at any point of time during isometric exercise using this exercise methodology together with fast-imaging MRI.

